# ALS Patient Stem Cells for Unveiling Disease Signatures of Motoneuron Susceptibility: Perspectives on the Deadly Mitochondria, ER Stress and Calcium Triad

**DOI:** 10.3389/fncel.2015.00448

**Published:** 2015-11-19

**Authors:** Anjoscha Kaus, Dhruv Sareen

**Affiliations:** ^1^Board of Governors-Regenerative Medicine Institute, Cedars-Sinai Medical CenterLos Angeles, CA, USA; ^2^Department of Biomedical Sciences, Cedars-Sinai Medical CenterLos Angeles, CA, USA; ^3^iPSC Core, The David and Janet Polak Stem Cell Laboratory, Cedars-Sinai Medical CenterLos Angeles, CA, USA

**Keywords:** ALS, motoneurons, *C9orf72*, *TDP43*, mitochondria, endoplasmic reticulum, calcium homeostasis, iPSCs

## Abstract

Amyotrophic lateral sclerosis (ALS) is a largely sporadic progressive neurodegenerative disease affecting upper and lower motoneurons (MNs) whose specific etiology is incompletely understood. Mutations in superoxide dismutase-1 (*SOD1*), TAR DNA-binding protein 43 (*TARDBP*/TDP-43) and *C9orf72*, have been identified in subsets of familial and sporadic patients. Key associated molecular and neuropathological features include ubiquitinated TDP-43 inclusions, stress granules, aggregated dipeptide proteins from mutant *C9orf72* transcripts, altered mitochondrial ultrastructure, dysregulated calcium homeostasis, oxidative and endoplasmic reticulum (ER) stress, and an unfolded protein response (UPR). Such impairments have been documented in ALS animal models; however, whether these mechanisms are initiating factors or later consequential events leading to MN vulnerability in ALS patients is debatable. Human induced pluripotent stem cells (iPSCs) are a valuable tool that could resolve this “chicken or egg” causality dilemma. Relevant systems for probing pathophysiologically affected cells from large numbers of ALS patients and discovering phenotypic disease signatures of early MN susceptibility are described. Performing unbiased ‘OMICS and high-throughput screening in relevant neural cells from a cohort of ALS patient iPSCs, and rescuing mitochondrial and ER stress impairments, can identify targeted therapeutics for increasing MN longevity in ALS.

## Introduction

Amyotrophic lateral sclerosis (ALS), or Lou Gehrig’s disease, is one of the most common neurodegenerative disorders and adult-onset motoneuron disease (MND), with a global incidence of 2.5 cases per 100,000 individuals each year, and is classified as a rare disease. The disease is primarily characterized by a relentless degeneration of upper motoneurons (UMNs) in the cerebral cortex and lower motoneurons (LMNs) in the brain stem and spinal cord, causes muscle weakness, progressive paralysis, speech and swallowing disabilities, and ultimately leads to death by respiratory failure (Al-Chalabi and Hardiman, 2013). A definite cause for ALS is not known except for a minority of cases with a familial history pointing to a genetic mutation as an origin (Table 1). ALS exists in two forms: heritable (familial, fALS) and idiopathic (sporadic, sALS), with the former featuring autosomal dominant or recessive inheritance patterns in 10% of total ALS cases and the latter represent the overwhelming majority of ALS cases (Cozzolino and Carri, 2012).

**Table 1:** **Gene mutations in clinical ALS**.

Clinical subtype	Locus	Gene	Onset/inheritance	Phenotype
***Removal of reactive oxygen species***
ALS1	21q22	Superoxide dismutase-1 (*SOD1*)	Adult/AD	Classical
***RNA binding and processing***
ALS4	9q34	Senataxin (*SETX*)	Juvenile/AD	Classical
ALS6	16p11.2	Fused in sarcoma (*FUS*)	Adult/AD	Classical
ALS9	14q11.2	Angiogenin (*ANG*)	Adult/AD	Classical
ALS10	1p36.2	TAR DNA-binding protein 43 (*TARDBP*)	Adult/AD	Classical
ALS13	12q24.12	ATXN2	Adult	Classical
***Endosomal trafficking and cell signaling***
ALS2	2q33	Alsin (*ALS2*)	Juvenile/AR	Classical
ALS11	6q21	Polyphosphoinositide phosphatase (Figure 4)	Adult/AD	
ALS8	20q13.3	Vesicle-associated membrane protein-associated protein B (*VAP-B*)	Adult/AD	Classical
ALS12	10p13	Optineurin (*OPTN*)	Adult/AD and AR	Atypical
ALS–FTD	9q21–q22	Chromosome 9 open reading frame 72 (*C9ORF72*)	Adult/AD	Atypical
***Glutamate excitotoxicity***
ND	12q24	d-amino acid oxidase (*DAO*)	Adult/AD	Atypical
***Ubiquitin/protein degradation***
ND	9p13–p12	Valosin-containing protein (*VCP*)	Adult/AD	Atypical
ALSX	Xp11	Ubiquilin 2 (*UBQLN2*)	Adult/X-linked	Classical
**Cytoskeleton**
ALS–dementia–PD	17q21	Microtubule-associated protein tau (*MAPT*)	Adult/AD	Atypical
***Other processes***
ALS5	15q15–q21	Spatacsin (*SPG11*)	Juvenile/AR	Classical
ALS–FTD	9p13.3	σ Non-opioid receptor 1 (*SIGMAR1*)	Adult/AD Juvenile/AR	Atypical
***Unknown processes***
ALS3	18q21	Unknown	Adult/AD	Classical
ALS7	20ptel–p13	Unknown	Adult/AD	Classical

*A list of genes associated with classical and atypical ALS. The left column groups the genes according to their cellular functions, the second to left column lists the corresponding chromosomal locus, and the middle column gives the gene name, if applicable. In the second to right column the time of disease onset and means of inheritance are displayed, while the right column links each gene to the disease phenotype in the clinic. AD, autosomal dominant; AR, autosomal recessive; FTD, frontotemporal dementia; PD, Parkinson’s disease*.

Since the discovery of the first gene responsible for familial forms of ALS, superoxide dismutase-1 (SOD1), fundamental advances in our understanding of the disease have been made with regard to biological, genetic and clinical processes (Cozzolino and Carri, 2012). Currently, ALS is considered a multi-factorial syndrome with motor system degeneration only being part of a more widespread disease process (Ilieva et al., 2009). Although, it is still not understood why particular populations of neurons are particularly vulnerable, increasing evidence over recent years emphasized that in addition to MNs, other cell types such as astrocytes, microglia, oligodendrocytes (OGs) and muscle cells may also actively participate in disease processes, such as neuroinflammation and muscle degeneration (Robberecht and Philips, 2013).

An ever-growing number of genes have been identified that are associated with ALS pathogenesis and account for a large fraction of familial cases of the disease, even in sporadic forms (Table 1). Interestingly, many of the same genetic mutations reported to be involved in familial and some sporadic forms of ALS have also been found to be associated with non-MN phenotypes, such as dementia in frontotemporal lobar degeneration (FTLD; Cooper-Knock et al., 2014), emphasizing a great overlap in the molecular pathogenesis of both disease entities (Goldstein and Abrahams, 2013).

In recent years the major focus of ALS research has moved to RNA related control of MN function and pathogenesis. Mutations in transactive response (TAR) DNA-binding protein (TARDBP or TDP43; Kabashi et al., 2008) and fused in sarcoma/translocated in liposarcoma (FUS/TLS, ALS6; Kwiatkowski et al., 2009; Vance et al., 2009) have been linked to fALS. The two proteins are involved in the regulation of RNA transcription, splicing, transport and translation and this enabled the establishment of new experimental models, both, *in vivo* and *in vitro* (Sreedharan et al., 2008; Vance et al., 2009; Zhou et al., 2010a). A more recently identified gene, *C9orf72*, is profoundly affected by expansions of GGGGCC (G_4_C_2_) repeats. *C9orf72* encodes for a protein with two different isoforms: (i) a mainly diffuse in the cytoplasm localized C9-L and (ii) a nuclear membrane localized C9-S (Xiao et al., 2015). The latter one shows redistribution to the cytoplasm of diseased MNs in ALS and an interaction with the nuclear pore complex components importin β1 and Ran-GTPase. The finding of reduced levels of at least one *C9orf72* transcript in expanded-repeat-carriers suggests a potential loss-of-function mechanism (Dejesus-Hernandez et al., 2011; Renton et al., 2011; Gijselinck et al., 2012; Donnelly et al., 2013; Sareen et al., 2013). Alternatively, the accumulation of transcripts containing the G_4_C_2_ transcripts as nuclear RNA foci are considered to confer the mutant gene with a toxic feature via an RNA-dependent gain of function mechanism (Dejesus-Hernandez et al., 2011; Achsel et al., 2013).

These three genes alone account for more than half of the reported fALS cases making RNA dys-metabolism one of the central issues of ALS pathogenesis. Several additional genes have been found to cause rare or atypical forms of fALS (Table 1). Nevertheless, based on their biological role and acquired cellular phenotypes, mutations in these genes have been also linked to oxidative stress, protein-misfolding and aggregation, endoplasmic reticulum (ER) and cytoskeleton alterations, ubiquitin proteasome pathway malfunctions, glutamate-mediated excitotoxicity, calcium (Ca^2+^) imbalance, and axonal transport defects (Cozzolino et al., 2012). Intriguingly, whether and how these recently described nuclear pore-mediated and RNA dysmetabolism-related pathways are intricately linked to oxidative stress pathways and mitochondrial damage phenotypes observed in many of ALS experimental models should be a focus of further studies.

**Figure 1:**
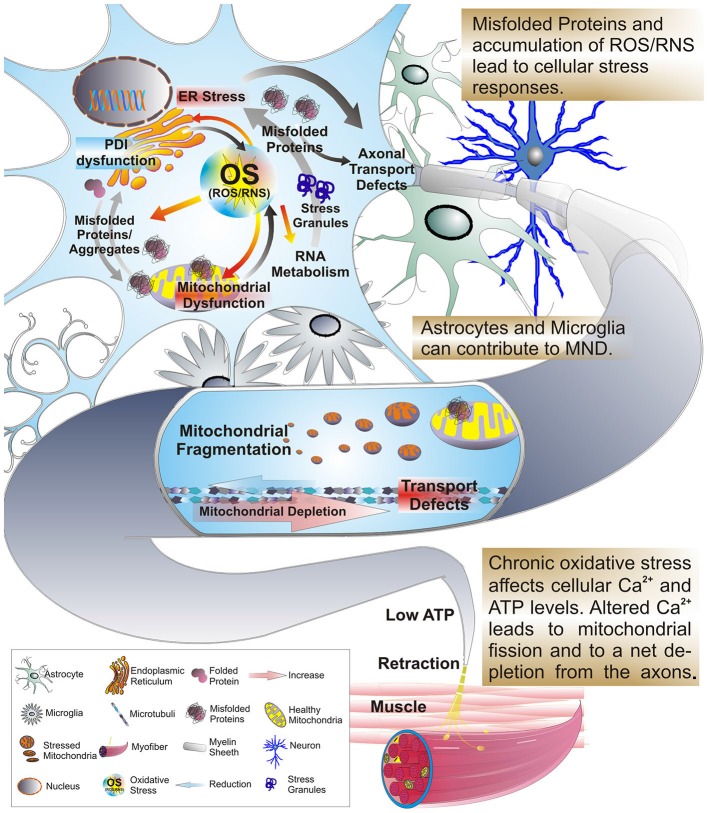
**Oxidative stress, protein misfolding and mitochondrial dysfunction are closely related.** Excessive production of reactive oxygen or nitrogen species (ROS/RNS), transcriptional dysregulation, protein misfolding and ER stress can arise as consequences of OS and mitochondrial stress. In addition these factors work in a feedback-loop further exacerbating mitochondrial stress and dysfunction. A significant amount of mitochondrial proteins, including those of the ETC, contain highly oxidizable iron-sulfur-clusters that upon exposure to OS can be severely affected in their folding and function. But, OS also triggers stress responses in other organelles, such as the ER and persistent stress and highly oxidative conditions impair the function and integrity of protein folding in the ER. As a result the formation of misfolded proteins is favored leading to an accumulation of insoluble cytosolic and mitochondrial aggregates, impaired interference with activity of the PDI and impaired axonal transport. During the course of these alterations the levels of ATP and intra-cellular calcium are affected. This change interferes with the Ca^2+^ and ATP sensitive mitochondrial fusion/fission machinery and microtubule based mechanisms of mitochondrial transport. Mitochondria accumulate in the cell soma in a fragmented and dysfunctional state leading to a dramatic reduction of mitochondria transported anterograde to the axon terminal. Given the size of motor neurons with long axonal extensions, the impaired axonal transport leads to a depletion of functional mitochondria at the axon terminal. With the axonal periphery no longer supplied with sufficient ATP distal synapses degenerate eventually and the MN dies. As a consequence myofibers no longer receive input from their corresponding MN and are prone to atrophy, manifesting in increased muscle weakness of ALS patients.

**Figure 2:**
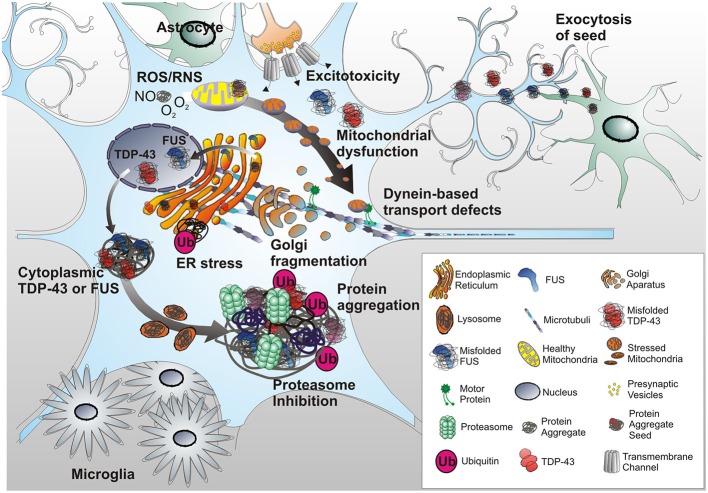
**Protein misfolding triggers ER stress in neurons.** Misfolded proteins trigger the unfolded protein response (UPR) of the ER, leading to cellular efforts in either refolding proteins to a normal state or ubiquitinating or degrading unrepairable proteins. Some proteins, i.e., TDP-43 or FUS escape degradation via proteasomes and autophagy resulting in large aggregates. Also, mutant TDP-43 and FUS accumulate in the cytoplasm. Continuous activation of UPR and other ER stress responses lead to ER dysfunction, fragmentation of the Golgi apparatus and protein trafficking defects. Adding to this, ROS and RNS released by ER and mitochondria, as well as excitotoxicity via glutamatergic synapses yield mitochondrial stress and fragmentation and further contribute to ER stress. As a result further formation of misfolded proteins is favored leading to an accumulation of insoluble cytosolic and mitochondrial aggregates, and an impaired activity of the PDI of the ER. Collectively, these events culminate with further overall neuronal dysfunction including dynein based transport inhibition. Ultimately, this results in a breakdown of energy supply for the distal axon terminal. Astroglial dysfunction or toxicity can contribute to MN degeneration as well, whether as initiating event or contributing factor remains to be determined. Activated astrocytes and microglia can trigger inflammatory responses resulting in further MN stress. Finally, a prion-like hypothesis posits that misfolded protein aggregates can spread from surrounding cells, either affected dying neurons or astrocytes, and infects MNs thereby initiating stress responses and ultimately apoptosis in the infected cells.

Mitochondria play a key role in cellular respiration by converting nutrients into ATP thereby providing cellular processes with energy. They are also the main source of reactive oxygen species (ROS) and act as gatekeepers in intrinsic apoptotic pathways. Mitochondrial dysfunction can lead to oxidative stress, failure of cellular bioenergetics and ultimately to cell death (Figure 1). Thus, an alteration of their properties could confer an intrinsic susceptibility to stress and aging of long-lived post-mitotic MNs in MNDs (Cozzolino et al., 2008; Cozzolino and Carri, 2012). ALS patient tissue and animal models exhibiting mitochondrial alteration and dysfunction have frequently been found to also exert ER stress. As the cellular compartment where secreted and membrane proteins are synthesized and folded, the ER is equipped with foldases, chaperones and co-factors to process these proteins and to prevent misfolding or aggregation. Stress conditions can interfere with ER function and result in abnormal folding and aggregation of proteins as has been observed in TDP-43 and FUS/TLS mutations (Andersen and Al-Chalabi, 2011; Turner et al., 2013) thus provoking a state of ER stress (Boillée et al., 2006a; Pasinelli and Brown, 2006; Matus et al., 2013; Figure 2). Given the large size and long neuritis of MNs, mitochondria and ER dysfunctions significantly interfere with their normal electrophysiological function as described in the following chapter.

Despite enormous advances in understanding the pathogenic events occurring in ALS, only a small fraction of all reported cases can be linked to genetic causes (Table 2). Thus the cause of sALS is largely unknown, and while environmental parameters have been a subject of many epidemiological studies (Seelen et al., 2014; Ingre et al., 2015; Malek et al., 2015), unidentified genetic factors are also thought to be a contributing factor towards susceptibility of MN degeneration. Considerable efforts have been made to unravel the genetic etiology of ALS with genome-wide association studies and other next-generation sequencing approaches (Renton et al., 2014; Cady et al., 2015). Whole exome sequencing studies, for instance, have identified *de novo* risk genes and pathways, i.e., TBK1 (Cirulli et al., 2015), thereby shedding new light on established ALS associated gene mutations and how they converge in pathways in fALS and sALS.

**Table 2:** **Animal models of ALS**.

ALS sub-type	Gene mutations	Animal models
Spontaneous motor neuron degeneration	Wobbler	**Mouse**—closely resembles hallmarks of clinical ALS, underlying mutation in Vps54 not identified in human ALS (Moser et al., 2013)
	Neuromuscular degeneration	**Mouse**—Strong neuromuscular degeneration phenotype, underlying mutation in Ighmbp2 was found in SMA patients (Van Den Bosch, 2011)
	Progressive Motor Neuronopathy	**Mouse**—Hindlimb paralysis and progressive motor axon degeneration, underlying gene product Tbce associated with human hypoparathyroidism retardation dysmorphism (HRD) syndrome (Van Den Bosch, 2011)
Mendelian fALS	SOD1	**Mouse/Rat**—Strong ALS-like phenotype in G93A, G37R, G85R, and D90A; variable severity across entity of SOD lines; H4R and G93A do not exert classical ALS; no TDP-43 abnormalities observed in many lines (Carri et al., 2015) **Worm**—Some of the cellular and molecular hallmarks of clinical ALS phenotypes (Therrien and Parker, 2014)
	FUS	**Mouse/Rat**—Some features of clinical ALS (Deng et al., 2014) **Fly**—recapitulates key features of ALS (Casci and Pandey, 2015) **Worm**—displays molecular MN degeneration phenotypes resembling ALS/FTLD (Therrien and Parker, 2014)
	TDP-43	**Mouse/Rat**—represents some features of clinical ALS, wide range of phenotype variation (some lines no MN phenotype), no clear aggregation or loss of nuclear TDP-43 (Chen-Plotkin et al., 2010; Tsao et al., 2012) **Zebrafish**—recapitulates some hallmarks of ALS, strong MN degeneration, cytoplasmic TDP-43 **Fly**—recapitulates some ALS aspects, strong MN degeneration documented, cytoplasmic TDP-43 (Casci and Pandey, 2015) **Worm**—MN degeneration phenotype (Therrien and Parker, 2014)
Atypical and rare mendelian ALS	ALS2/Alsin	**Mouse**—A very mild, considerably subtle phenotype; no major MN deficits; loss of cerebellar Purkinje cells (Cai et al., 2005)
	VAP-B	**Mouse**—VAP-B (P56S) mutants do not show signs of MN degeneration; cytoplasmic TDP-43 accumulations in MN and spinal cord (Van Den Bosch, 2011)
	Dynactin P150^glued^	**Mouse**—Axonal transport defects; accumulation of cytoskeletal and synaptic vesicle proteins at NMJ, loss of spinal motor neurons, increase of astrogliosis (Van Den Bosch, 2011)
Genetic abnormality in fALS and sALS	C9ORF72	**Mouse**—Motor deficits and loss of cortical and cerebellar neurons similar to clinical symptoms of ALS/FLTD; RNA-foci, TDP-43 pathology; astrogliosis (Rohrer et al., 2015) **Zebrafish, fly** and **worm**—MN degeneration, more detailed studies awaiting (Therrien and Parker, 2014; Casci and Pandey, 2015)

*A representation of some of the vertebrate and invertebrate models utilized to study the cellular pathology of MND. The left column group’s spontaneous mutations in animal models found to closely resemble MND pathology, Mendelian and atypical forms of ALS associated mutations. In the middle column disease associated genes are listed. The right column summarizes how far ALS-associated phenotypes resemble the designated models*.

In this review, we will outline the main factors that have been associated with ALS disease progression and pathology, the impact of organelle dysfunction underlying dysfunction in MNDs. Mitochondrial dysfunction and ER stress have been implicated as major events in MN degeneration and we will highlight findings from animal and cell-based models and how those contribute to degenerative phenotypes of neuronal and non-neuronal cells. With an increasing number of studies utilizing patient-derived induced pluripotent stem cells (iPSCs) to recapitulate disease phenotypes we will outline some of the observed phenotypes and to what extent they resemble hallmarks of disease progression observed in the clinic.

## Motoneurons Involved in ALS

The risk of developing ALS peaks between 50–75 years of age, which is in contrast to other neurodegenerative diseases such as Parkinson’s disease (PD) or Alzheimer’s disease (AD), thus emphasizing that it is not necessarily a disease of aging, but that age is one of the chief risk factors. In a majority of patients, ALS manifests as asymmetric, painless weakness in a limb, referred to as spinal onset. About 20% of patients present with symptoms of weakness in bulbar muscles, difficulties swallowing (dysphagia) and speech impairment, referred to as bulbar onset. In about 3–5% of patients, ALS is characterized by onset affecting the respiratory system, with shortness of breath and difficulty breathing (orthopnea and dyspnea). While these symptoms of onset can be overlapping, the average survival is 2–5 years, with bulbar and respiratory onsets having a worse prognosis (Swinnen and Robberecht, 2014).

MN diversity is generated during CNS development by members of the *hox* gene family and an array of inductive signaling pathways. Although, it is not yet completely understood how different subtypes of MNs are specified during development, work over the past decades has significantly advanced our understanding of *hox* genes conferring regional identity to MN groups along the rostro-caudal axis (Philippidou and Dasen, 2013). With over 300 bilateral pairs of muscles comprised of over a 100 million muscle fibers, over 120,000 MNs in the spinal cord alone account for sensation of muscle, contraction, respiration and other vital functions. Upper MNs (UMNs) that are affected in ALS originate either in the motor region of the cerebral cortex or in the brainstem and carry motor information down to the lower MNs (LMNs). LMNs are either located in the anterior gray column and anterior nerve roots—termed spinal LMNs, or the cranial nerve nuclei of the brain stem and cranial nerves with motor function, summarized as cranial nerve MNs. Voluntary movements rely on spinal LMNs, which innervate skeletal muscle fibers, thereby acting as a link between UMNs and muscles. In turn, cranial nerve LMNs control movement of the eyes and tongue as well as contributing to swallowing, chewing and vocalization (Swinnen and Robberecht, 2014).

Based on the type of muscle LMNs innervate, they can be further classified into alpha-, beta-, and gamma MNs. Alpha MNs (αMNs) innervate extrafusal muscle fibers, the most abundant type of muscle fiber and the type being involved in muscle contraction. Beta MNs (βMNs) innervate intrafusal fibers of muscle spindles with collateral extensions to extrafusal slow twitch fibers. Gamma MNs (γMNs) innervate intrafusal muscle fibers and together with sensory afferents, compose a muscle spindle and are part of proprioception, the sensing of body positioning. According to the contractile properties of the motor units (MU) they form with their target muscles, αMNs can be further classified into fast-twitch fatigable (FF), fast-twitch fatigue-resistant (FR) and slow-twitch fatigue-resistant (SR; Burke et al., 1973).

### Subtypes of MNs are Differently Affected in ALS

Not all MN subtypes are affected equally in ALS. In SOD1 transgenic mice the FF MU undergo atrophy the earliest, with a nearly total loss of FF terminals from muscle fibers in the triceps area (Frey et al., 2000). The almost synchronous dieback is followed by delayed, but likewise rapid fallout of the FR units, while S MU remain well preserved until late in disease progression (Pun et al., 2006). Electrophysiological studies and fiber type analysis confirm the overall sequence of degeneration; however, they also suggest an initial switch in MU phenotype from FF to FR to precede the loss of FF axons (Kieran et al., 2005; Hegedus et al., 2008; Gordon et al., 2010). The relative resistance observed in FR and S MU may by explained be their high sprouting capacity, which could enable them to innervate motor endplates that have been vacated by FF axons (Frey et al., 2000). In accordance with this electromyogram (EMG) patterns reflect cycles of denervation/reinervation (de Carvalho et al., 2008) and the FF MU being affected first in sALS patients as well (Dengler et al., 1990).

A selective vulnerability of particular neuronal subsets to the neurodegenerative process is a key feature, not only of ALS, but of other neurodegenerative diseases. Why certain subtypes of MNs are particularly susceptible to injury in the presence of mutations affecting ubiquitously distributed proteins such as TDP-43 and SOD1, is not well understood. The cell-specific features of LMNs, i.e., a large cell size, long axons and large terminal arbors, have great implications for their energy metabolism, the requirement of mitochondrial and cytoskeletal support, and the distribution of mRNA for protein synthesis (Ferraiuolo et al., 2011). LMNs vulnerable to injury in ALS exhibit particular sensitivity to glutamatergic toxicity via a-amino-3-hydroxy-5-methyl-4-isoxazole propionate (AMPA) receptor activation. Unlike other neuron groups, they exert high expression levels of calcium-permeable AMPA receptors that lack the GluR2 subunit (Williams et al., 1997) and have fairly low amounts of cytosolic calcium-buffering proteins (Ince et al., 1993). In addition these MNs have a high threshold for initiating a protective heat-shock protein response, an increased sensitivity to ER stress (Saxena et al., 2009) and mitochondrial features that predispose them to calcium overload and oxidative damage (Sullivan et al., 2004; Panov et al., 2011).

Data obtained in ALS patients and animal models alike initially suggested an early axonal dieback and selective susceptibility of FF axons to occur during human disease progression (Fischer and Glass, 2007). With a selective dieback of FF MU in ALS, intrinsic molecular mechanisms conferring susceptibility to these units lie at hand. In case of the many different point mutations reported for mutant SOD1, research has mainly focused on how misfolded SOD1 toxicity impacts the cell. For instance excessive accumulation of misfolded proteins leads to stress responses in the ER in an effort to restore cellular homeostasis. When this process is not successful, as is the case in SOD1 mutants, ER pathways can trigger apoptosis (Matus et al., 2013). Interestingly, a recent study targeting mSOD1 in UMN via an AAV9-shRNA in a presymptomatic SOD1 (G93a) rat model reported significant delay of disease onset and improved survival of spinal MNs (Thomsen et al., 2014). These findings suggest differential vulnerability of MN subtypes alongside early UMN dysfunction during the presymptomatic phase of disease course.

With the recognition of non-neuronal cells such as glia and activated microglia also have a major contribution to the progression and outcome of the disease three scenarios have been hypothesized: (I) The “dying forward” hypothesis suggests that events within the MN are the primary determinant initiating MN damage, while alterations in closely associated non-neuronal astrocytes, Schwan cells, microglia and skeletal muscle cells only contribute to the disease progression; (II) In a counter-proposed “dying backward” mechanism, ALS is considered to begin within the muscle cells and/or neuromuscular junction as a result of one or more neurotrophic factors being released by postsynaptic cells. When retrogradely transported via the presynaptic axon to the cell body those factors may initiate MN damage and degradation (Dupuis and Loeffler, 2009); and (III) Alternatively, the “prion-like propagation” scenario could explain the focal onset and propagation of misfolded ALS protein seeds via the neuronal network or the extracellular matrix (Polymenidou and Cleveland, 2011).

## Contribution of Other Cell Types to ALS

### GABA-Ergic Interneurons

During the last 20 years, structural and functional neuroimaging studies have greatly revised our longstanding notions of the pathophysiology of ALS. In fact, anatomical lesions extend beyond precentral cortices and corticospinal tracts, and include the corpus callosum, the frontal, sensory and premotor cortices, thalamus and midbrain. Theories of glutamate excitotoxicity and hyperexcitability in ALS suggest the contribution of excessive synaptic excitation, for instance through AMPA receptors (Kwak et al., 2010) and Na^+^ channels. Disease-specific changes in RNA editing of the AMPA glutamate receptor subunit 2 (GluR2), for instance, have been found in spinal MNs of human sALS autopsy cases (Kawahara et al., 2004). Clinical data, however, suggest the possibility of insufficient synaptic inhibition. Magnetic resonance imaging (MRI) and positron emission tomography (PET) studies in patients revealed an early and diffuse loss of inhibitory cortical interneurons and diffuse gliosis in white matter tracts (Chió et al., 2014). In fact, MN excitability is strongly modulated by synaptic inhibition mediated by presynaptic glycinergic and GABAergic innervations and postsynaptic glycine receptors (GlyR) and GABA_A_ receptors (Martin and Chang, 2012).

Abnormal glycine and GABA levels have been observed in ALS patients (Malessa et al., 1991; Niebroj-Dobosz and Janik, 1999; Eisen and Weber, 2000). In human ALS autopsy samples of the spinal cord, glycine binding sites are reduced in the anterior horn and in human ALS autopsy motor cortex (Hayashi et al., 1981; Whitehouse et al., 1983). In agreement with this, mRNA levels of the alpha1-subunit of the GABA receptor of inhibitory interneurons was significantly reduced, while the GABA synthesizing enzyme glutamic acid decarboxylase (GAD), in turn, was dramatically upregulated in the prefrontal and temporal cortices of ALS patients (Petri et al., 2006). In the spinal cord of adult transgenic G93A-hSOD1 transgenic mice (Schutz, 2005), as well as organotypic spinal cord-slice cultures of embryonic G93A-hSOD1 transgenic mice (Avossa et al., 2006), signs of imbalance excitatory and inhibitory innervations, indicative of aberrant neuroplasticity, are evident. Thus aberrant or even failed inhibition may be involved in ALS mechanisms of disease.

### Glia—Microglia

The immune system plays a crucial role in the maintenance of tissue homeostasis and the response to injury or infection. Within the central nervous system (CNS) microglia, the major resident immune cells, constantly survey the microenvironment and produce factors that influence other glial cells such as astrocytes. While microglia remain in a deactivated state during physiological conditions (Streit, 2002) they switch to an activated state upon pathogen invasion or tissue damage detected by their Toll-like receptor 4 (TLR4) and promote inflammatory responses via mediators such as TNF-α or IL-1β, engaging the immune response and tissue repair (Saijo et al., 2009). Usually this response is self-limiting and is resolved when infection has been eradicated or tissue damage has been repaired. Sustained inflammation, however, leading to tissue damage implies persistent inflammatory stimuli or failure in resolution mechanisms. A persistent stimulus can either result from environmental factors or chronic persistence of endogenous factors, being perceived by the immune system as “stranger” or “danger” signals and the subsequent inflammatory responses may overwhelm normal resolution mechanisms. While some inflammatory stimuli produce beneficial effects, i.e., phagocytosis, uncontrolled inflammation may as well result in generation of neurotoxic factors that amplify the underlying disease states (Glass et al., 2010).

Signs of neuroinflammation can be readily observed in pathologically affected areas in the brain and spinal cord in both human ALS patients as well as mouse models of the disease. Characteristic for ALS are gliosis and accumulation of large numbers of activated microglia and astrocytes marked by elevated production of inflammatory mediators (i.e., COX-2), proinflammatory cytokines (i.e., IL-1β, IL-6 and TNF-α) and potentially cytotoxic molecules such as ROS. For instance, major histocompatibility complex molecules and complement receptors were found highly expressed by activated microglia in the primary motor cortex and the anterior horn of the spinal cord in ALS patients (McGeer and McGeer, 2002). Receptors of the innate immune response could be potential sensors of molecules that induce or amplify inflammation in ALS (Letiembre et al., 2009), given that CD14, a protein facilitating TLR4 responses to lipopolyssaccharides (LPS), and TLR2 are upregulated in the spinal cords of mouse models (Nadeau and Rivest, 2000; Nguyen et al., 2001) and ALS patients (Letiembre et al., 2009; Liu et al., 2009). Indeed, a study conducting chronic infusion of presymptomatic ALS mice with LPS documented an enhanced innate immune response and exacerbated disease progression (Nguyen et al., 2004). Another piece of evidence for an active involvement of microglia in disease progression is the finding that mutant SOD1, but not wild-type protein, is presumably secreted into the extracellular space via chromogranin vesicles, resulting in activation of microglia and MN death in culture (Urushitani et al., 2006). Consistent with this is the toxicity of ALS patient derived CSF for exposed rat spinal cord cultures and the presence of mSOD1 within this CSF (Tikka et al., 2002) and intracerebral infusion of mutant SOD1 into wild-type mice can induce microglia activation and cytokine production (Kang and Rivest, 2007).

### Glia—Astrocytes

Even though MNs are the primary cells affected in ALS, accumulating evidence points to a role of supporting astroglia during pathogenesis. MNs isolated from transgenic mSOD1 mice were found to be more sensitive to Fas or NO triggered cell death than in wild-type neurons suggesting a MN specific death pathway (Raoul et al., 2002). Binding of the ligand FasL to the Fas receptor leads to its intracellular portion recruiting the adaptor protein FADD which in turn activates a caspase cascade that culminates ultimately in MN death. While this pathway was found activated in presymptomatic ALS mice as well (Raoul et al., 2006), it is noteworthy that mSOD1 astroglia produce significant NO and FasL (Barbeito et al., 2004), thus implying that astroglia could in fact be the executioner of MN death in ALS. In line with this, the p75 neurotrophin receptor (p75^NTR^) has been associated with ALS-associated MN death, as nerve growth factor (NGF) secreted by mSOD1 astrocytes induced death of p75^NTR^ expressing MNs via the formation of NO and peroxide (Pehar et al., 2004).

Cell-specific targeting of mSOD1 deletion in astrocytes and microglia utilizing GFAP-cre and CD11b-cre transgenes, respectively, was found to reduce severity of the disease phenotype and dramatically improve survival of ALS mice (Boillée et al., 2006b; Yamanaka et al., 2008b). While concerns related to the timing and efficiency of gene targeting in these mice have been raised, additional supporting evidence for an involvement of non-neuronal cells comes from studies with chimeric mice selectively expressing mSOD1 in MNs and non-neuronal cells. In such chimeras the presence of wild type non-motor neurons significantly delayed the onset of MND despite a high penetrance of mSOD1 MNs and OGs, increasing disease free life by 50% (Yamanaka et al., 2008a), and in co-cultures mSOD1 astrocytes exerted toxic effects on healthy wild-type MNs (Di Giorgio et al., 2008; Marchetto et al., 2008). These experiments suggest a non-cell-autonomous scenario, for SOD1-meidated ALS at least, in which sick or mutant astrocytes directly contribute to MN degeneration.

That non-neuronal cells have a crucial impact on MN degeneration is also supported by the finding of mutant protein aggregates in neighboring astrocytes (Zhang et al., 2008). In a series of elegant experiments dramatic impact of ALS associated TDP-43 mutations were evident in human astrocytes. The authors generated a significantly purified population of astrocytes from patient-derived iPSCs with specific TDP-43 mutations (M337V) characterizing the cellular phenotype and found the astrocyte cultures to accumulate 30% more cytoplasmic TDP-43 than control astrocytes. Control-astrocytes that were transfected with mutant TDP-43 in turn displayed a similar amount of cytoplasmic TDP-43 accumulation (Serio et al., 2013). This emphasizes a direct responsibility of the specific TDP-43 mutation in the observed subcellular mislocalization.

### Glia—Oligodendrocytes

OGs are of particular importance for neuronal function and axonal transmission as they insulate axons with an essential myelin sheath. This myelin sheath allows for the fast and efficient propagation of action potentials and neuronal electrical transmission over long distances through saltatory conduction (Nave, 2010a,b; Simons and Lyons, 2013). Apart from this, OGs are a principal supplier of metabolic energy to axons and neurons.

Until recently, the involvement of OGs and their precursors in the pathogenesis of ALS was fairly unexplored. Abnormalities of OGs, such as pathological inclusions immunoreactive for smooth muscle alpha-actin, but neither angiotensin nor TDP-43, were observed in affected post mortem tissue of ALS patients. Pathological aggregates sequestering the ALS-linked protein TDP-43 occur in the cytoplasm of OGs from both sporadic and familiar ALS patients (Seilhean et al., 2009; Murray et al., 2011; Philips et al., 2013). Another ALS-linked protein, FUS, was found sequestered into cytoplasmic inclusion of patients with FUS-linked ALS. Interestingly, patients with late onset of FUS-linked ALS are characterized by highly abundant FUS inclusions in OGs, while early onset patients preferentially have neuronal cytoplasmic inclusions (Mackenzie et al., 2011; Nonneman et al., 2014).

Studies on ALS-linked mSOD1 transgenic mice revealed that gray matter OGs in the spinal cord degenerate before the first symptoms of MN degeneration become apparent (Kang et al., 2013; Philips et al., 2013). Indicatory for this OG degeneration is an abnormal morphology manifested in thickened, irregularly shaped cell body, enlarged cytoplasm and elongated reactive processes. Such dysmorphic OGs are in an apoptotic state characterized by cleaved caspase-3 immunoreactivity and altered chromatin condensation (Kang et al., 2013; Philips et al., 2013) and are significantly increased in SODG93a mice already before disease onset and are progressively increasing throughout (Philips et al., 2013). In addition, dense clustering of reactive microglia around degenerating OGs can be observed (Kang et al., 2013). Lineage tracing of OGs with a tamoxifen-inducible OG-specific Cre line in mSOD transgenic mice (*Plp*-*CreER*; *Rosa26*-EYFP, *SOD1*^G93A^) confirmed the progressive decrease in the number of OGs as a function of disease progression in SOD1^G93A^ mice (Kang et al., 2013; Philips et al., 2013). Morphological examination, however, revealed dysmorphic somata and branches and altered lipid and protein composition of the myelin sheath. Besides myelin defects, the newly generated OGs also fail to supply neurons with metabolic energy substrates, including lactate, pyruvate and ketone bodies (Philips et al., 2013). In addition, activated microglia and astrocytes exert OG toxicity as microglia for instance produce ROS and secret pro-inflammatory cytokines (Nonneman et al., 2014).

**Figure 3:**
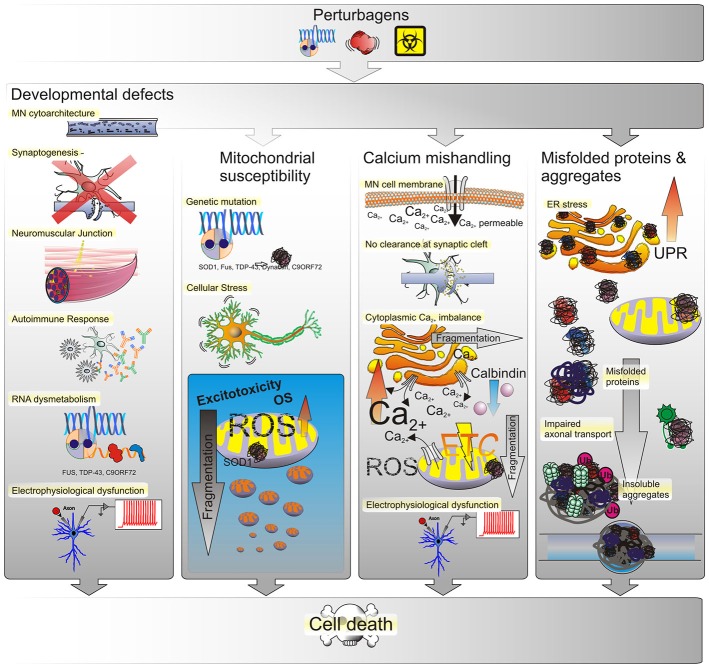
**Possible causes of ALS.** The most prevalent underlying reason for MN defects are genetic perturbagens, inherited or *de novo* mutations. Yet, the majority of ALS cases have not been linked to any mutation, suggesting that other effectors such as the environment may play into this as well. Thus MN phenotypes observed in ALS may arise from genetic and/or environmental perturbagens, as depicted in the top panel. Developmental anomalies may affect the structural integrity of neuronal cytoarchitecture as conferred by structural proteins, transport proteins, transmembrane proteins or by a disruption of RNA processing. Such defects can interfere or even prevent the formation of synapses between neurons or at the NMJ either directly within MN or indirectly by affecting neighboring glial cells. Triggering of autoimmune responses and chronic low-level inflammation may lead to MN degeneration as well and many of those developmental defects manifest in electrophysiological deficiencies such as a progressive decrease in voltage-activated Na^+^ and K^+^ currents correlated with a loss of functional outputs. Mitochondrial susceptibility due to ROS-induced OS in turn yields an inert vulnerability of MNs to excitotoxicity. An increased amount of mitochondrial stress in turn leads to mitochondrial fragmentation and ultimately cell death. Due to their large size and long neurite outgrowths, MNs are particularly sensitive to ion fluctuations, whether due to selective permeability for Ca^2+^ influx or lack of messenger clearance from the synaptic cleft. The dysregulation of intracellular Ca^2+^ levels has severe implications for MN function as well. Both, the ER and mitochondria function as buffers of cellular Ca^2+^ homeostasis. When intracellular Ca^2+^ levels increase, either by influx from the extracellular space or the ER and mitochondria, this triggers OS responses, their fragmentation and eventually progression of cell death signals that ultimately lead to loss of electrophysiological outputs. The generation of misfolded proteins and formation of aggregates, likewise affects the functional integrity of both organelles. Initially, misfolded proteins trigger the UPR in the ER to compensate for decreased protein translation and processing efficiency. Persistence of misfolded proteins that escape corrective degradation mechanisms cause accumulation in the cell and ultimately lead to the formation of insoluble aggregates interfering with cellular functions such as molecular transport and trafficking.

Although inflammation may not necessarily represent an initiating factor in neurodegenerative diseases, intriguing evidence obtained both from patient tissue and from animal models over the recent years stressed that sustained inflammatory responses involving microglia, astrocytes and OGs contribute to disease progression (Figure 3). This is the case not only in ALS and FTLD but in many other neurodegenerative diseases such as Alzheimer’s, Parkinson’s and multiple sclerosis (Glass et al., 2010). Whether inhibition of sustained inflammatory responses can be a safe and successful means to reverse or to at least slow down the course of disease progression should be a focus of further investigations. Disease-in-a-dish models utilizing ALS patient iPSC-derived co-cultures of MNs with astrocytes or OG could be used to study whether pharmacological inhibition of inflammation in the latter two cell-types would improve MN survival *in vitro*. As a second step such astrocytes and OG with pharmacological inhibition of inflammation could be transplanted into the CNS of ALS disease animal models to evaluate whether reducing inflammation in non-neuronal cells *in vivo* would ultimately slow MN degeneration or even improve MN health. Should this be the case, it will point to inflammation being the key contributor to trigger mitochondrial dysfunction, Ca^2+^-imbalance and ER stress responses.

**Figure 4:**
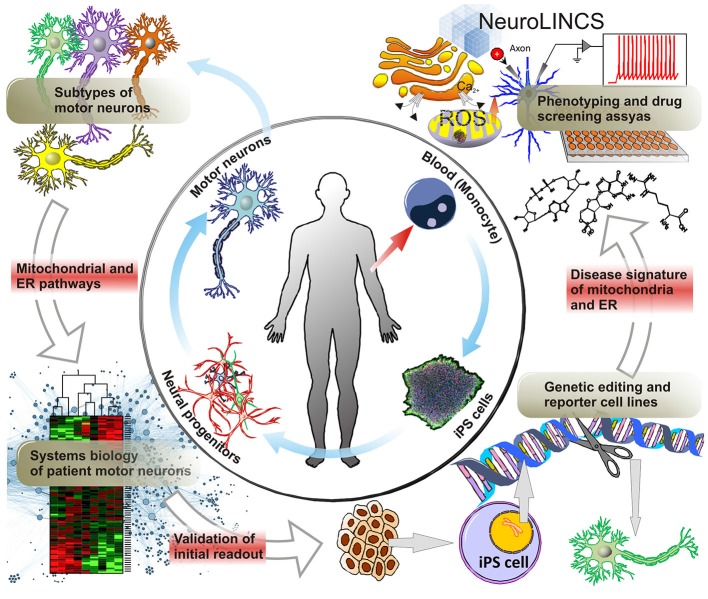
**Molecular disease signatures of MNDs.** ALS Patient derived cells, i.e., skin cells or whole blood allow for the generation of iPS cells and the subsequent differentiation into MNs via neural progenitors. Not only does iPS cell technology now allow for the large scale production of MNs but, conceptually, also the generation of all the diverse subtypes of upper and lower MNs affected in ALS. As a disease-in-a-dish model, patient-iPSC-derived motor neuron subtypes can aid in unraveling the molecular mechanisms involving mitochondrial and ER dysfunction, as a cause of ALS vs. being a secondary effect after disease onset. Systems biology approaches utilizing large scale genomics, transcriptomics and proteomics will help identify novel targets of MNDs such as ALS and SMA compared to iPSC derived MNs from control patients. For further analysis and validation of prominent gene targets, gene editing techniques creating reporter and genetic loss-of-function cell lines can facilitate the development of disease signatures of ALS patient iPSC-derived MNs. Particularly, collaborative efforts such as i.e., the NeuroLINCS initiative will enable the acquisition of large data sets of cellular phenotypes and drug screening assays. Building on established data from animal models this approach may dramatically accelerate the discovery of valid new therapeutic approaches and drug targets.

## Mitochondrial Involvement in ALS

Mitochondria perform diverse functions, such as producing ATP via oxidative phosphorylation, and regulating cellular levels of metabolites, amino acids and co-factors for various regulatory enzymes. As a main source of ROS and buffer of spatiotemporal Ca^2+^ homeostasis, they integrate signals with other cellular compartments and contribute to cellular stress responses such as autophagy and apoptosis (Nunnari and Suomalainen, 2012). Further, mitochondrial fission and fusion specifies health in metabolic disorders and neurodegenerative diseases (Chen and Chan, 2009; Westermann, 2010).

The unique cytological properties of MNs, such as their extended cell size and the large volume of the axonal compartment, require a high metabolic demand and crucially depend on a robust cytoskeleton and axonal transport mechanisms. Due to that, MNs show increased generation of ROS and a tendency towards oxidative stress, making them particularly prone to other factors, i.e., ER stress, altered calcium levels and excitotoxicity (Figure 1). Developmental defects due to genetic mutations may confer mitochondria with an inert susceptibility towards developing MND (Ferraiuolo et al., 2011) as discussed.

### Structural Alterations

In samples of ALS patients an altered ultrastructure is evident, with swollen and vacuolated mitochondria found in MNs, muscles, and intra-muscular nerves (Afifi et al., 1966; Atsumi, 1981; Siklos et al., 1996; Sasaki et al., 2007). Axonal swellings in MNs, Bunina bodies, comprised of accumulated neurofilaments, organelles and secondary lysosomes have been observed in the remaining LMNs and constitute a hallmark of ALS-specific histopathology (Hart et al., 1977; Okamoto et al., 1990). Imaging experiments on MNs derived from ALS transgenic mSOD1 mice revealed a reduced mobility (Magrane et al., 2009) and a net increase in retrograde transport of mitochondria away from the axon terminal, resulting in a depletion (De Vos et al., 2007, 2008).

A common feature of mitochondrial morphology changes in cellular and animal models of ALS is fragmentation of the mitochondria. Expression of mSOD1 in neuronal cells *per se* induces mitochondrial fragmentation. This pattern of alteration in mitochondrial morphology is for instance observed when interfering with the expression of aptic atrophy 1 (OPA1), a pro-fusion factor, dynamin related protein 1 (Drp1; Ferri et al., 2010) and other factors such as PINK1 (Matsuda et al., 2010; Deas et al., 2011). While mitochondrial alteration has been extensively studied in mSOD1 animal models, it remains to be determined, whether such changes are consistently found in other ALS associated mutations, both in animal models and human stem cell derived MNs.

### Reactive Oxygen Species (ROS) and Oxidative Stress

Mitochondria are particularly vulnerable to oxidative stress given that: (i) excessive ROS leads to lipid peroxidation interfering with mitochondrial membrane integrity; (ii) a plethora of mitochondrial proteins, such as essential proteins of the ETC complexes, contain highly oxidizable iron-sulfur clusters; and (iii) due to the lack of protective histone complexes mitochondrial DNA is far more susceptible to mutations in organelle encoded proteins (Cozzolino et al., 2012). Thus, conditions that interfere with mitochondrial homeostasis or lead to mitochondrial dysfunction are self-sustaining due to increased ROS production and accumulation. Experimental evidence obtained in mouse models have the focus of other reviews (Cozzolino et al., 2012).

Localized oxidative stress in ALS mitochondria, specifically ETC impairments, has been documented in patients and SOD1 mice (Kong and Xu, 1998; Miana-Mena et al., 2005). This was accompanied by energy deficits in the spinal cord (Jung et al., 2002; Mattiazzi et al., 2002; Kirkinezos et al., 2005), primary astrocytes and primary motor cortex (Cassina et al., 2008; Loizzo et al., 2010). A reduction in mitochondrial repair enzymes in patients and SOD1 mice (Kikuchi et al., 2002; Murakami et al., 2007), and markers of oxidative stress such as lipid peroxidation, protein glycoxidation and altered protein carbonyl levels in patient samples (Shibata et al., 2002) emphasize an exacerbating mitochondrial dysfunction in ALS (Mahoney et al., 2006). In mSOD1 mice phenotypes manifest in muscles with hypermetabolism, reduced adipose tissue accumulation and elevated energy expenditure already in the asymptotic phase (Dupuis and Loeffler, 2009; Dupuis et al., 2009). Such findings, however, have not been reported in patients and other ALS models, raising the question whether the early oxidative stress associated muscle phenotype in SOD1 animal models is an ALS specific early phenotype or, instead, rather a sub-form of ALS, specific to mutations in SOD1, and finally, whether such phenotypes may only be specific to the SOD1 animal models themselves.

### Calcium Buffering and Homeostasis

MNs express a large number of Ca^2+^ permeable receptors lacking the GluR2 subunit (Greig et al., 2000; Heath et al., 2002; Van Damme et al., 2002; Kawahara et al., 2003), and in addition to that, have particularly low Ca^2+^ buffering abilities (Lips and Keller, 1998; Palecek et al., 1999). This could make MNs particularly sensitive to glutamate toxicity exerted through AMPA receptors, however would mostly contribute to disease severity in later stages (Grosskreutz et al., 2010; Cozzolino and Carri, 2012). In turn selective loss of the astroglial glutamate transporter GLT1 is a more likely scenario of neurotoxicity due to impaired glutamate clearance from the synaptic cleft (Rothstein et al., 1995; Bendotti et al., 2001; Howland et al., 2002).

Toxic Ca^2+^ dysmetabolism in ALS is probably mostly evoked by a deficiency in intracellular Ca^2+^ mishandling, as a consequence of low levels of Ca^2+^-binding proteins or impaired mitochondrial Ca^2+^ buffering. Supporting this, calbindin D-28K immunoreactivity was found reduced in the frontal cortex of patients with FTLD (Ferrer et al., 1993), and in ALS patients, with significant differences in the densities of calbindin positive neurons within cortical layers V and VI (Maekawa et al., 2004). A loss of calbindin and parvalbumin immunoreactivity from subsets of MNs in presymptomatic mSOD1 mice suggests calbindin loss as an early event in ALS pathogenesis (Sasaki et al., 2006).

Evidence for defective Ca^2+^ storage as a result of mitochondrial dysfunction initially came from cell models for SOD1-linked ALS, which displayed elevated cytoplasmic Ca^2+^ levels in conjunction with compromised mitochondrial gradient potential (Carri et al., 1997; Jaiswal et al., 2009). In animal and cell models of mSOD1, MNs expressed markers of oxidative stress and mitochondrial dysfunction, elevated Ca^2+^ levels and vulnerability to glutamate toxicity (Kruman et al., 1999). Further *in vivo* studies manifested prolonged Ca^2+^-induced membrane polarization in an early decrease in mitochondrial Ca^2+^ loading capacity, impaired Ca^2+^ uptake in neurons and muscle tissue (Damiano et al., 2006; Jaiswal and Keller, 2009; Nguyen et al., 2009; Zhou et al., 2010b). Adding to this it has been found that Ca^2+^ is able to bind to SOD1 immature states and promote its aggregation (Leal et al., 2013). This emphasizes that, apart from mutations in ALS-related proteins disrupting calcium homeostasis, Ca^2+^ itself affects ALS-critical proteins and cellular processes as well.

In summary, Ca^2+^ dysregulation and mitochondrial pathology are tightly interconnected. Apart from SOD1, however, many other ALS-critical proteins have been shown to potentiate Ca^2+^ dysregulation thereby increasing cellular Ca^2+^ vulnerability as well (Leal and Gomes, 2015). Altered Ca^2+^-influx can increase ROS production and accumulation and cytochrome c release, making this process self-sustaining and steering towards activation of cell death (Dykens, 1994). Although it is tempting to speculate that Ca^2+^ could be the one factor shifting MN integrity out of balance, future studies will need to address early Ca^2+^ phenotypes further. Intriguingly, Ca^2+^ dysregulation and mitochondrial pathology are also intimately connected to protein aggregation as we will discuss in the next chapter.

### Non-SOD1 Mutation Derived Mitochondrial Alteration and Damage

In recent years, a number of studies have described mitochondrial alteration and dysfunction in non-mutant-SOD1-linked cases of ALS. TDP-43, a protein with dual functions as DNA-binding protein with nuclear export sequence and as RNA-binding protein, is found to be predominantly localized to the cytosol instead of the nucleus in ALS spinal MNs, found in punctate immunoreactive granules and as dense skeins (Kabashi et al., 2008). Transgenic mice overexpressing a human TDP-43 (hTDP-43) under the control of the mouse prion protein reporter in the brain and spinal cord, produce an ALS-like phenotype exhibited by reactive gliogenesis, axonal and myelin degeneration, gait abnormalities and early lethality. The hTDP-43 overexpression induces abnormal perinuclear aggregates of mitochondria accompanied by enhanced levels of fission and fusion components, Fis1, phosphorylated Dlp1 and mitofusin 1, respectively (Xu et al., 2010). Accumulation of mitochondria in TDP-43-negative cytoplasmic inclusions in MNs was also found in a *Thy1.2*; *hTDP-43* mouse model that notably lacked mitochondria in motor axon terminals. This, however, could be due to an alteration in elements of the transport machinery, kinesin-associated proteins Kif3a and KAP3, found in inclusions (Shan et al., 2010). Alternatively, it has been observed that TDP-43 stabilizes the RNA of human low molecular weight neurofilament protein via interaction with the 3^′^UTR, altering its location in spinal MNs and thereby possible favoring the formation of NF aggregates in ALS (Strong et al., 2007) and mitochondria mis-localization.

The RNA-binding protein FUS/TLS is normally localized in the nucleus where it participates in process involved in RNA maturation, among those snRNP biogenesis, alternative splicing, and mRNP localization. Most of the research on the role of mutant FUS in ALS has focused on its mislocalization and aggregation, and little is known about its involvement in mitochondrial dysfunction. Mutant forms of FUS/TLS were found to accumulate in the cytoplasm, similarly to TDP-43, and form protein inclusions in spinal horn neurons derived from ALS patients (Deng et al., 2010). In a few juvenile ALS cases post-mortem tissue analysis of MNs revealed basophilic inclusions containing abnormal aggregates of FUS proteins and disorganized intracellular organelles, among those mitochondria and endoplasmic reticuli (Huang et al., 2010).

While the SOD1 model may only represent a small fraction of the heterogeneous disease population and posits a note of caution when interpreting mSOD1 mouse phenotypes at large across idiopathic forms of ALS, recent findings supporting the view of OS and mitochondrial damage to play a role in non-SOD1 ALS also come from cell and invertebrate models (Carri et al., 2015). The phenotypes found in mutant TDP-43 and FUS/TLS support the concept of mitochondrial dysfunction and damage as a major event in ALS progression. Further investigation is needed to elucidate if mitochondrial alteration and dysfunction phenotypes in non-SOD mutants occur at early disease stages as a key gateway in ALS pathogenesis.

### Mitochondrial Susceptibility as Cause vs. Result of ALS

While ALS-associated mutations can to some extent explain the circumstances leading to ROS and Ca^2+^ dysregulation in MN ultimately resulting in apoptosis, they do not account for the overwhelming majority of sALS cases in which no genetic inheritance has been found. A limited ability of MNs to balance intracellular Ca^2+^ levels, however, may very well be attributable to sALS cases as well. Given the large cytoarchitecture of MN axons this raises the question whether mitochondrial phenotypes in ALS could be a matter of inert susceptibility being an early event in the onset of MN degeneration. Other than heritable genetic mutations of fALS cases, factors interfering with mitochondrial function and Ca^2+^ homeostasis, such as environmental perturbagens and/or yet to be identified modifier genes, could provoke prolonged mitochondrial stress and affect cell function in sALS cases. Compelling evidence exists pointing to approaches that artificial manipulation and rescue of mitochondrial dynamics may lead to new therapeutic approaches that would, to the least, extend MN life and improve disease conditions in patients (Chen and Chan, 2009).

### Screening and Reversing Mitochondrial Phenotypes

A major hurdle in clinical progression of ALS still is that the time for a definite diagnosis of patients, which may take 15 months or longer. Given an average life expectancy of 3–5 years, it is crucial to develop faster means of clinically detecting ALS. As a complex disease with a wide range of phenotypical presentation, in the clinic as well as in animal models, mitochondrial stress and dysfunction represent a common feature of MN degeneration (Talbot, 2014) and may proof valuable as molecular markers in future diagnostics. Rescuing mitochondrial dysfunction in ALS ultimately represents a valuable target for many other neurodegenerative diseases as well. Human stem cell derived disease-in-a-dish models could provide additional insight. Patient-derived fALS iPSCs have been shown to reproduce some of the neuronal stress and degeneration phenotypes (Donnelly et al., 2013; Sareen et al., 2013; Kiskinis et al., 2014; Wainger et al., 2014). *In vitro* screening in human MNs and reversing associated mitochondrial dysfunction signatures may thus represent an invaluable means of unraveling molecular signatures of human neurons.

## Endoplasmic Reticulum (ER) Stress

The ER is the primary compartment in which secreted and membrane proteins are processed to ensure proper folding via different classes of folding mediators. A cohort of molecular chaperones assists the folding process of unfolded or misfolded proteins preventing aggregation. Such chaperones are found in multiple sub-cellular compartments, and each of those compartments possesses its own subset of chaperones (Zapun et al., 1999). A number of stress conditions can interfere with the normal function of the ER leading to abnormal oxidative folding of proteins at the ER lumen inducing ER stress (Boillée et al., 2006a; Pasinelli and Brown, 2006; Matus et al., 2013). Mutations in ALS disease causative genes, such as TARDBP/TDP-43, FUS/TLS, and vesicle-associated membrane protein-associated protein B (VAP-B), for instance, trigger aggregation of the mutant proteins leading to a gain of neurotoxic activity and provoking ER stress (Andersen and Al-Chalabi, 2011; Turner et al., 2013). These conditions engage the UPR, a signal transduction pathway responding with increased protein folding capacity and quality control mechanisms of the ER to restore homeostasis (Hetz et al., 2011; Walter and Ron, 2011). Chronic ER stress, however, yields irreversible damage of the neuronal cells and ultimately MN death (Hetz, 2012).

Three major stress sensors activate the UPR. (1) The inositol-requiring transmembrane kinase/endonuclease (IREI1), the PKR-like ER kinase (PERK), and activating transcription factor 6 (ATF6). IREI1 initiates splicing of an mRNA coding for the transcription factor X-Box-Binding protein 1 (XBP1), converting it into a potent activator of multiple UPR-responsive genes, the XBP1s (Calfon et al., 2002). XBP1s in turn control the expression of genes responsible for protein folding, protein quality control, and secretion or ER-associated degradation (ERAD; Acosta-Alvear et al., 2007), while IREI1 further engages in degradation of specific subsets of RNAs and pathways involving Janus Kinases (JNK), mediating apoptosis and autophagy levels (Ron and Walter, 2007). (2) PERK is a stress sensor and once activated, reduces protein translation into the ER by phosphorylation of the eukaryotic initiation factor 2 alpha (eI2Fα), leading to a decrease in misfolded protein overload (Harding et al., 1999). The eI2Fα enables expression of the gene ATF4, which in turn upregulates a cascade of UPR-targeted genes responsible for amino acid and redox metabolism, protein folding, autophagy, and apoptosis. One of those target genes, CHOP, is a crucial mediator of apoptotic processes in response to ER stress, supposedly via driving the expression of pro-apoptotic Bcl-2 family members, such as BIM and PUMA (Tabas and Ron, 2011; Walter and Ron, 2011), in addition to GADD45. Sustained PERK activation, however, contributes to apoptosis by increasing oxidative stress and resuming protein synthesis after prolonged ER stress (Marciniak et al., 2004; Verfaillie et al., 2012; Han et al., 2013). (3) The third major stress sensor, ATF6, is activated in the ER and translocates to the Golgi apparatus. Following processing, its released cytoplasmic domain acts as a transcription factor (Walter and Ron, 2011). The activated ATF6 in turn controls certain sets of target genes related to protein folding and quality control (Haze et al., 1999; Chen et al., 2002).

### Misfolded Proteins and Protein Aggregates in Motoneuron Disease

Familial ALS mutations have been associated with an accumulation of misfolded proteins (Andersen and Al-Chalabi, 2011; Turner et al., 2013), increased ER stress and dysfunction as well as pathogenic structural alterations of the ER in ALS patient tissue (Oyanagi et al., 2008; Sasaki, 2010; Vijayalakshmi et al., 2011). In human postmortem tissue of sALS cases ER stress was evident by distended and fragmented ER cisternae in affected cells of the anterior horn of the spinal cord. Ribosome-free membranous structures extending from the ER and electron dense material resembling Bunina bodies, Hirano bodies and honeycomb-like structures were observed in samples as well (Oyanagi et al., 2008; Sasaki, 2010; Vijayalakshmi et al., 2011). Interestingly, a recent study immunolabeling patient tissue with GRP78 for BiP expression found even normal appearing MNs labeled and display dilated ER lumen with amorphous and granular material in electron microscopy analysis (Oyanagi et al., 2008). However, many technical limitations exist in interpreting causative processes and the precise kinetics in many cellular neuropathological observations made in post-mortem ALS tissue.

TDP-43, a member of the heterogeneous nuclear ribonuclearprotein (hnRNP) subclass of 2xRBD-Gly proteins, shuttles between the nuclear and cytoplasmic compartments, binding both DNA and RNA, and is involved in exon splicing of several genes (Chen-Plotkin et al., 2010). In both ALS and FTLD pathogenesis TDP-43 was found as the most frequent component of ubiquitinated protein inclusions. The clinical histopathology is characterized by cytoplasmic inclusions of skein-like or dense granular appearance and by clearance of TDP-43 from the nucleus (Geser et al., 2008). TDP-43 can bind to RNA in a single-stranded and sequence-specific manner, required for many RNA processes, and its most C-terminal portion bears unique characteristics making it prone to misfolding and aggregation (Buratti and Baralle, 2008; Johnson et al., 2009; Achsel et al., 2013; Al-Chalabi and Hardiman, 2013).

Expansions of a hexanucleotide G_4_C_2_ repeat in the first intron/promoter region of the *C9ORF72* gene were identified as the most frequent genetic cause of both f/sALS cases and FTLD (Dejesus-Hernandez et al., 2011; Renton et al., 2011; Gijselinck et al., 2012). Reports suggested that the repeat is transcribed and leads to accumulation of repeat-containing RNA foci in patient’s tissues (Dejesus-Hernandez et al., 2011). Studies using stem cell models of ALS, one by the Sattler and Rothstein groups and another by our group (Donnelly et al., 2013; Sareen et al., 2013), generated iPS cells from patients with ALS caused by C9ORF72-associated repeat expansion. While patient-iPSC derived MNs were not adversely sensitive to cell death processes, they exhibited certain electrophysiological phenotypes. The mutant C9ORF72 transcript containing G_4_C_2_ repeat expansions conferred a gain of function neurotoxicity, which was abolished upon correcting gene expression profiles by anti-sense oligonucleotides (ASOs) introduction (Donnelly et al., 2013; Sareen et al., 2013). C9ORF72 mutant animal models, with one exception (Koppers et al., 2015), reported to recapitulate key molecular signatures unique to the intronic G_4_C_2_ repeat expansion, including RNA-foci and inclusions of dipeptide repeat (DPR) expansion inclusions, TDP-43 pathology, and behavioral abnormalities similar to symptoms observed in C9ORF72/ALS patients (Chew et al., 2015). Furthermore, some of these studies were able to associate C9ORF72 repeat expansions to interfere with nucleocytoplasmic shuttling and identified elements of the nuclear import/export machinery to either enhance or suppress DPR mediated toxicity in Yeast and fly models (Freibaum et al., 2015; Jovicic et al., 2015; Zhang et al., 2015).

Many fALS mutation occur in genes encoding key components of protein quality control. This includes components essential for the retrograde transport of autophagosomes from axons to the cell body such as dynein and dynactin (Moughamian and Holzbaur, 2012; Ikenaka et al., 2013), the autophagic adaptor p62 (Fecto et al., 2011), as well as the UBA-containing proteins Ubqln2 and Optineurin (Fecto and Siddique, 2012). Elevated levels of ER chaperones, foldases, cell death signals and upregulation and activation of the IREI1, PERK and ATF6 branches of UPR signaling have been reported by multiple studies in sALS patient spinal cord tissue (Ilieva et al., 2007; Hetz et al., 2009; Ito et al., 2009). Misfolded proteins are constantly generated in many compartments of the cells, and are usually removed by quality control systems composed of the ubiquitin (Ub)-proteasome system (UPS), chaperone mediated autophagy (CMA) and macroautophagy. Proteins that escape the surveillance of the UPS and CMA or tend to form aggregates are subjected to macroautophagy for degradation into amino acids (Hariharan et al., 2011; Koga and Cuervo, 2011; Ciechanover and Kwon, 2015). SOD1 and TDP-43 may additionally perturb their folding, leading to the formation of β-sheet enriched aggregates (Andersen and Al-Chalabi, 2011) that are resistant to all known proteolytic pathways and can further grow into inclusion bodies and extracellular plaques (Figure 2; Koga and Cuervo, 2011).

Aggregating proteins form intracellular inclusions containing Ub and Ub ligases, as found in animal models (Bruijn et al., 1997; Bendotti et al., 2004) and patient tissue (Leigh et al., 1991; Bendotti et al., 2004; Sasaki, 2010) becoming visible in the brain stem and spinal cord at onset of the manifestation of ALS symptoms (Watanabe et al., 2001). Although these large inclusions are clinical hallmarks of ALS symptoms, they are unlikely to be toxic to neurons. In fact they may be a neuroprotective phenomenon, as monomeric and oligomeric misfolded ALS proteins seem to be toxic to MNs (Guo et al., 2010). Monomeric and oligomeric misfolded proteins in turns may exert the actual toxicity in ALS according to recent findings suggesting a “prion-like” focal propagation of the disease (Polymenidou and Cleveland, 2011; Sugaya and Nakano, 2014). In support of that mutant and wild-type SOD1 have been found to spontaneously fibrillize (Grad et al., 2011; Munch et al., 2011) and both TDP-43 and FUS were proposed to contain a prion-like glutamine/asparagine (Q/N)-rich domain found in inclusions. *In vitro* these inclusions exhibited ordered, self-perpetuating aggregation and were transmissible from one cell to its progeny (Ito et al., 2011; Polymenidou and Cleveland, 2011).

Despite an ever growing number of ALS-causative genes having been identified, yet, difficult to identify one key factor driving disease progression in MNs. Protein misfolding and the effects of mono- and oligomeric forms on sequestering functional RNAs and proteins from cellular processes constitute a plausible cause of MND and are in alignment with many of the ALS typical hallmarks found in ALS post mortem tissue. Given the sensitivity of RNA and protein metabolism, rapid development of disease phenotypes (i.e., TDP43, C9orf72) are often observed in animal models such as mice and flies. The corresponding genetic mutations in ALS patients however, display later in life with median onset age greater than 50. Many more elements of ER stress responses, i.e., the UPR, have been found to impact phenotypes of animal models and reviewed elsewhere (Matus et al., 2013).

### Screening Assays for Testing and Reversing UPR Phenotypes

A number of studies have shown that ER chaperones can be secreted into the extracellular space upon cellular stress (Dorner et al., 1990). In line with this is the finding of PDIA1 levels being upregulated in the cerebrospinal fluid (CSF) of ALS patients (Atkin et al., 2008) and a study exposing spinal MNs to CSF of ALS patients documented an induction of ER stress in the MNs (Vijayalakshmi et al., 2011). Furthermore proteomic biomarkers in blood samples of ALS patients revealed the up-regulation of chaperones, such as the ER-stress responsive chaperones PDIA1 and ERp57. This was also confirmed for mononuclear cells from blood of mSOD1 mouse models. Finally, in a longitudinal study TDP-43, cyclophillin A, and ERp57 were strongly associated with disease course in ALS patients and control subjects (Nardo et al., 2011).

UPR signaling responses convert information about the nature and intensity of the occurring stress into a network of partially overlapping target genes that orchestrate an adaption to the stress, or trigger cell death programs as a last resort. Given the need for biomarkers to quantitatively assess disease stage, prognosis and efficacy of clinical trials, these studies suggest measuring stress factors released into the CSF or the blood as a valuable tool for diagnosis and monitoring ALS disease progression. Regardless of the origin of cellular stress in MNs, it is increasingly clear that modulating stress due to protein (mis-)folding and the proteostatic capacity of MNs represent a promising therapeutic target to delay the symptomatic phase of ALS. With regard to this, utilizing gene therapy or small molecule approaches to reinforce the capacity of coping with ER stress mechanisms may be an applicable means for disease intervention (Matus et al., 2013). While animal models of ALS provide valuable insight into many aspects of genetically linked disease progression *in vivo*, there are, however, limitations to these mouse models with regard to specificity of the human disease phenotypes (Table 3). To this end, ALS patient-derived iPSCs and fluorescent reporter cell lines can serve as an important complementary tool to monitor and discover molecular markers of ER stress and UPR, specifically in different subclasses of MNs from patients bearing the patient genotype.

**Table 3:** **Hallmarks of ALS in animal models**.

Clinical ALS	Animal models of ALS						
phenotypes	SOD1	Wobbler	FUS	TDP-43	ALS2	P150glue	C9ORF72
Macroscopic hallmarks	(M) ±	(M) +	M (+)	(M) ±	M (−)	(M) ±	(M) +
Degeneration of upper and lower MNs	+	+LMN	+	+LMN	−	+	+
	+ MN		+LMN	MN			+
			±	MN			+
				±			
Vesicle traffic defects	+	+	+	+	ND	+	+
	ND						
Enlarged endosomes vacuolization	+	+	+	+	ND	+	+
	±						
Impaired axonal transport	+	+	+	+	+	+	
				+			
Protein mis-sorting	G93A	+			ND	+	
Ubiquitin-positive protein aggregates	G93A	+	+	+	ND	ND	+
	±		±	±			
TDP-43-positive protein aggregates	(−)	+	+	±	ND	ND	+
	±		±	±			
Neurofilament aggregations (perinuclear)	G93A	+	ND	ND	ND	ND	
	Res		±	±			
Mitochondrial alteration	G93A	+	ND	+	ND	ND	
	ND						
Cortical	+	+	+	ND	+	ND	
hyperexcitability/excitotoxicity	ND		ND				
Astrogliosis	+	+	+	+	−	+	+
Microgliosis	+	+	+		−	+	+
NMJ and Muscle atrophy	+	+	+	±	±	+	+
			+	+			
	±		ND	+			
				±			
Hyperexcitability, reduced GABAergic inhibition	G93A	+	+	+			
			ND

*Cellular and molecular features presenting in clinical ALS are listed in the far left column. Some of the most frequently found mutations found associated with fALS and sALS are represented at the top and the phenotypes found in animal models summarized for each category. Color coding: Black, mouse/rat; Red, fruit-fly; Green, worm; Blue, zebrafish; +, phenotype; −, no phenotype; ±, variable phenotype among different animal lines; Res, restricted phenotype; ND, not determined (yet)*.

## Human Stem Cell Disease Models for ALS

The majority of disease processes involve multiple cell and tissue types and in principle, are best modeled utilizing animals. Not surprisingly, animal models have been instrumental to our understanding of human disease pathology, including neurodegenerative diseases such as PD, AD and ALS. Nonetheless, many neurodegenerative diseases, such as those affecting MNs, in fact do not naturally occur in commonly used laboratory animals. Introduction of certain pathogenic aspects of human neurodegenerative diseases with mutant genes being wildly overexpressed into animals often creates phenotypes that only partially resemble the original human disease (Table 2).

Introduction of mSOD1 into mice to induce disease phenotypes that resemble those manifested in patients requires expression of multiple copies of mSOD1. In human patients, a single copy is sufficient (Bruijn et al., 1997). Moreover, the most prevalent form of mSOD1 in ALS patients, the alanine to valine substitution (A4V), does not appear to generate phenotypes in mice (Furukawa et al., 2006). Thus, differences do exist between humans and mice, with the pathogenic potential of mSOD1 just being one example, that may complicate the study of some mutations in animal models alone. Astrocytes have also been found to be very different between humans and rodents and could add a potential caveat in disease modeling (Oberheim et al., 2009). However, as illustrated in the previous chapter, glial cells such as astrocytes have a critical role in the pathogenesis of ALS and other neurodegenerative diseases. As a consequence, the utilization of model systems with a human background as means of complementing studies in animal models can greatly benefit our understanding of MNDs.

### hESCs and iPSCs as Disease-In-A-Dish Models

In order to establish human disease models, there are two main routes of approach. The first approach is to genetically alter human stem cells with the target gene(s) of interest, and the second is to derive stem cells from patients with target diseases. The genetic modification of mouse embryonic stem cells (ESCs) by targeting specific gene loci via homologous recombination has been routinely applied in many laboratories as a first step to creating transgenic animals. Likewise, this same principle is applicable when using human embryonic stem cells (hESCs; Zwaka and Thomson, 2003). Repeated cellular cloning, however, as is required by traditional approaches using homologous recombination, can often render the established hESC cell line unstable. Alternatively, random gene insertion, by lenti-viruses for instance, can reduce cloning cycles significantly, yet transgenes that are integrated during the stem cell stage may be downregulated during differentiation (Xia et al., 2007). When Du et al. (2009) screened for gene loci resistant to gene silencing using a lentiviral vector with built-in Cre-loxP cassette, they were able to establish cell lines that were resistant to gene silencing even upon differentiation of these hESCs into neurons and astrocytes. These master cell lines in turn allow for the introduction of any gene of interest, including genes provoking human diseases, via the built-in Cre-loxP cassette through Cre recombination with high efficiency. In addition, recent technological advances allowed for the use of zinc fingers to target specific gene loci with high efficiency (Hockemeyer et al., 2009), alleviating the necessity to screen large numbers of cell clones.

The second approach, obtaining human stem cells with disease traits capturing the patient genotype, has been made available ever since the hallmark discovery that human induced pluripotent stem cells (hiPSCs) can be generated from somatic cells such as fibroblasts (Takahashi et al., 2007; Yu et al., 2007; Hanna et al., 2010). Somatic cells can be transitioned into iPSCs exhibiting phenotypes very similar to hESCs by expressing pluripotency factors such as Oct3/4 and Sox2 combined with either Klf4 and c-Myc, or Lin28 and Nanog (Takahashi et al., 2007; Yu et al., 2007). With similar approaches hiPSCs were generated from somatic cells harvested from patients with MNDs including ALS (Dimos et al., 2008; Table 4) and SMA (Ebert et al., 2009; Sareen et al., 2012). These hiPSCs can be differentiated can be readily differentiated into neurons and astrocytes.

**Table 4:** **Human stem cell models of ALS**.

	Human stem cell models	
Mendelian fALS	SOD1	**hiPSCs**—Strong ALS-like phenotypes in MNs, including neurite and axonal degeneration, protein aggregation and mitochondrial alterations, some TDP-43 abnormalities observed (Yang et al., 2013; Kiskinis et al., 2014; Chen et al., 2014)
	FUS	**hiPSCs**—Mutant FUS-P525L forms cytoplasmic aggregates in MNs (Wainger et al., 2014; Liu et al., 2015)
	TDP-43	**hiPSCs**—ALS MND phenotype variations, excessive MN death vs. enhanced vulnerability and dendrite alterations; TDP-43 proteinopathy and cytoplasmic aggregates, astrogliosis (Bilican et al., 2012; Egawa et al., 2012; Serio et al., 2013; Tsao et al., 2012)
Genetic abnormality in fALS and sALS	C9ORF72	**hiPSCs**—Increased MN susceptibility; presence of abnormal RNA foci and accumulation of non-ATG-dependent translation, TDP-43 pathology (Zhang et al., 2015; Sareen et al., 2013)

*An overview of human stem cell models utilized to study the cellular pathology of MND *in vitro*. The left column Mendelian and atypical forms of ALS associated mutations. In the middle columns representative model terminology and disease associated genes are listed and the right column summarizes in how far ALS associated phenotypes are resembled in the designated models*.

Many of the disease phenotypes such as MN death, or glial cell impairment occur in iPSC derived differentiated cells (Table 5), emphasizing that patient hiPSCs may provide a versatile model to dissect the cellular and molecular mechanisms underlying MN degeneration (Table 4). A recent study by Zhang and colleagues utilizing patient derived mSOD1 iPSCs (D90A and A4V) as a model for MN degeneration in ALS found mutant SOD1 to exhibit neurofilament aggregation specifically in MNs (Chen et al., 2014). This was accompanied by decreased stability of NF-L mRNA and binding of its 3^′^UTR by mSOD1, yielding altered protein proportion in NF subunits. Expression of a single copy of mSOD1 could mimic this MN-selective phenotype in hESCs as well, while a genetic correction of mSOD1 in patient-derived iPSC MNs could revert the phenotype. Interestingly, conditional NF-L expression in the mSOD1 iPSC derived MNs corrected the NF-subunit proportion, mitigating NF aggregation and neurite degeneration in MNs. This suggests an mSOD1 mediated misregulation and aggregation of NF heavily impacts axonal degeneration in ALS MNs (Chen et al., 2014). Moreover, two other studies using patient derived mSOD1 iPSC models reported transcriptional and functional changes in mitochondrial and ER stress pathways as causes of perturbed electrochemical activity in ALS neurons (Kiskinis et al., 2014; Wainger et al., 2014).

**Table 5:** **Phenotypes of human stem cell models of ALS**.

Clinical ALS phenotypes	Human stem cell models of ALS			
	SOD1	FUS	TDP-43	C9ORF72
Macroscopic hallmarks	+	+	±	+
Degeneration of upper and lower MNs	+	+	±	+
Vesicle traffic defects	ND	ND	ND	ND
Enlarged endosomes vacuolization	+	ND	ND	ND
Impaired axonal transport	ND	ND	ND	ND
Protein mis-sorting	+	ND	ND	+
Ubiquitin-positive protein aggregates	+	+	+	+
TDP-43-positive protein aggregates	ND	ND	+	+
Neurofilament aggregations (perinuclear)	+	ND	+	+
Mitochondrial alteration	+	ND	ND	+
Cortical hyperexcitability/excitotoxicity	ND	+	ND	ND
Astrogliosis	ND	ND	+	+
Microgliosis	ND	ND	ND	ND
NMJ and muscle atrophy	Neurite and	ND	Neurite and	Neurite and
	axonal degeneration	ND	axonal degeneration	axonal degeneration
Hyperexcitability, reduced GABAergic inhibition	+	+	+	+

*Cellular and molecular features presenting in clinical ALS are listed in the far left column. Some of the most frequently found mutations found associated with fALS and sALS are represented at the top and the phenotypes found in iPSCs summarized for each category. +, phenotype; −, no phenotype; ±, variable phenotype; ND, Not determined (yet)*.

Noteworthy, the studies above were also able to analyze patient iPSC-derived MNs harboring repeat expansions in *C9ORF72* and retrieve a subset of the transcriptional changes found in the mSOD1 iPSCs, indicating these are being broadly conserved in ALS (Kiskinis et al., 2014; Wainger et al., 2014). Other studies revealed disease specific phenotypes, i.e., dysregulated gene expression, G_4_C_2_ RNA foci, and susceptibility to excitotoxicity via a gain of function RNA toxicity (Donnelly et al., 2013; Sareen et al., 2013). These phenotypes could be mitigated with antisense oligonucleotides (ASO) targeting the *C9ORF72* transcript or repeat expansions despite the presence of repeat-associated non-ATG (RAN) translation products (Donnelly et al., 2013). RAN translation can express homopolymeric expansion proteins in all three reading frames, without an AUG start codon. This non-canonical type of protein translation stands in stark contrast to the classical rules of translational initiation, is length- and hairpin-dependent, and occurs without frameshift or RNA-editing as a result of the *C9ORF72* G_4_C_2_ repeat expansion (Zu et al., 2011; Cleary and Ranum, 2013). Interestingly, C9ORF72 mutant iPSC-derived MNs displayed activation of ER stress pathways and MN death when subjected to the ER specific stressor tunicamycin (Donnelly et al., 2013).

TDP-43 patient iPSC-derived mutant MNs formed *de novo* cytosolic aggregates similar to those observed in postmortem tissue of ALS patients (Egawa et al., 2012; Burkhardt et al., 2013). Not only was this observed consistently in independent studies, but iPSC derived mutant TDP-43 MNs were also shown to recapitulate the same degenerative phenotype as observed in postmortem tissue of the corresponding patient (Burkhardt et al., 2013). Moreover, the TDP-43 iPSC derived MNs exhibited shorter neurites and increased amount of mutant TDP-43 protein in a detergent insoluble from Egawa et al. (2012). Recently, mutations of TDP-43 in patient iPSC-derived MNs associated with reduced levels of micro-RNA 9 (miR-9) and its precursor pre-miR-9-2, suggesting miR-9 downregulation to be a potential common event in ALS and FTLD (Zhang et al., 2013). A recent study in turn reported an initial hyperexcitability followed by a loss of functional output and synaptic activity, yielding a progressive decrease in voltage activated Na^+^ and K^+^ currents (Devlin et al., 2015). Interestingly, this was observed in, both, mutant TDP-43 and C9ORF72 patient-derived MNs, suggesting an early defect in ion channel status as a common event contributing to potential initiation of downstream degenerative pathways in ALS.

Taken together, familial ALS patient-iPSC models recapitulate some of the disease phenotypes also observed in patient tissue and some of transgenic animal models. Human iPSC disease-in-a-dish models complement other established models to decipher the molecular pathways involved in ALS. Also, human iPSC-based models allow for greater versatility in screening of human ALS disease signatures by: (a) providing accessibility of difficult-to-obtain CNS cell types such as MNs; (b) allowing investigations on developmental effects of any ALS-associated mutation; (c) giving options to analyze single mutant cells; (d) supplying glial cells for interaction studies in co-culture with neurons; (e) allowing prospects of ALS biomarker discovery; and (f) providing platforms for therapeutic screening strategies.

### Identification of Molecular Markers

A crucial step on the way to treatment for ALS is the identification of an early biomarker signature. Whether it comprises markers of neural and glial pathogenesis, mitochondrial and ER stress signals (Cozzolino et al., 2012; Hetz, 2012), or early signatures before actual disease onset, such means of diagnosis could greatly facilitate determination of disease progression and the most applicable measures of treatment. To yield good biomarkers ALS models need to resemble the main characteristics of the disease phenotype in patients and allow for easy accessibility of the involved cell types. Animal models have been most frequently employed for this, with rodent animals such as mouse and rat being a major source, but even invertebrate models such as flies and worms (Carri et al., 2015).

Patient-derived iPS cells from a large cohort of well-phenotyped patients are another promising tool as they allow direct study of ALS patient-derived human cell types. For instance iPSC-derived MNs and glial cells could be screened for secreted stress molecules found in the CSF and blood of ALS patients (Atkin et al., 2008; Nardo et al., 2011; Vijayalakshmi et al., 2011). Likewise, in addition to animal models, pre-clinical models could incorporate ALS patient iPSC-derived cells in the discovery and testing of promising therapeutics. Such approaches have been put to test using patient iPSCs. In a chemical screen referring to the TDP-43 aggregate endpoint, a recent study identified FDA-approved small molecule modulators such as Digoxigenin, emphasizing the feasibility of patient-derived iPSCs-based disease modelling for drug screening (Burkhardt et al., 2013). Microarray analysis of iPSC-MNs from mutant TDP-43 patients, decreases in the expression of genes encoding cytoskeletal proteins and small increases in genes involved in RNA metabolism was observed. When treating the MNs with anacardic acid, a histone acetyltransferase inhibitor, they were able to rescue the abnormal MN phenotype (Egawa et al., 2012). Over the next 3–4 years there will be increasing accessibility of large cohorts of patient-derived iPSCs from thousands of ALS patients as a result of collaborative initiatives across the USA, in part due to funds raised by the ALS Ice Bucket Challenge, such as the TRACK/ENROLL-ALS, NeuroCollaborative, and Answer ALS among many others. These ALS iPSC panels bear great potential for screening and identification of robust biomarkers signifying various stages of ALS. In addition, iPSC-derived neurons and glia may be used to identify large sets of markers of diverse stressors thereby enabling the creation of an ALS disease signature.

There are significant limitations and challenges associated with patient iPSC-derived models. Some of these include: (a) obtaining homogenous population of neural subtypes devoid of undifferentiated progenitors; (b) prolonged time required in culture for maturation; and (c) fetal/young “age” of the different neural subtypes. Further development of cell sorting tools, fluorescent reporters, and better markers (surface/intracellular) for the human cell types is imperative. Recent advancements in genome editing and creation of CRISPR/Cas edited reporter iPSC lines will help solve some of these technical issues. The big question that remains and needs addressing is whether it is required to generate completely homogenous cellular models (neurons or glia) to mimic human ALS-in-a-dish. The answer is going to be context and question-dependent. Nevertheless, a motor neuron during development will not engender synaptic maturity without the presence of astrocytes in culture.

### iPSC Derived Cell Transplantation Approaches

Apart from using patient-derived iPSCs for identification of diagnostic markers, they may also bear the potential for cell-based therapies. While such approaches have been used in neurological diseases with focal pathology, i.e., PD (Björklund and Lindvall, 2000), replacement of affected or lost cells in widespread areas of the CNS, as encountered in ALS will prove to be very challenging. However, replacement of affected cells in critical parts of the brain and spinal cord, such as respiratory centers, on the other hand, may be life-saving.

The progression of ALS is heavily affected by interactions between MNs and neighboring glial cells, as outlined in the previous chapter. Thus, replacing diseased or toxic astrocytes at an early stage could potentially rescue MNs from degeneration. Replacement of astrocytes in support of MN health has been successfully conducted in a rat model of ALS. The primary astrocytes not only survived the transplant, but also contributed to increased animal life-span with grafted astrocytes migrating a certain distance along the spinal cord and offering support for MNs in the transplant site and neighboring areas (Lepore et al., 2008). Although the human spinal cord is of course substantially larger than that of rodent animals, it is nonetheless surgically feasible transplant cells at multiple sites. As dramatically enriched and even pure populations of human glial progenitors differentiated from PSCs in a chemically defined culture system can be produced in large quantities, production of clinical grade human astrocytes in a clean facility is also technically feasible. Specifically, generating astrocytes from the patient’s own somatic cells through reprogramming to iPSCs will circumvent the issues of immune rejection in transplantation (Liu and Zhang, 2010). We have also shown feasibility of transplantation and integration of iPSC-derived neural progenitor cells (iNPCs) cells into the rodent spinal cord. These iNPCs could be a promising therapeutic strategy for ALS. In the host environment, they differentiate into astroglia and provide the possibility of replacing lost cells, modulating the injury environment, and creating a permissive milieu to protect and regenerate host tissue (Sareen et al., 2014).

Replacement of diseased MNs via transplantation of iPSC derived neuronal cells remains very challenging. Although MN differentiation protocols from PSCs have not achieved pure populations, recent improvements are moving this field in the right direction (Maury et al., 2015). Clinical applications require xenobiotic-free reagents such as replacement of animal-derived protein growth factors with defined small molecules for the differentiation procedure. Moreover, grafted human MNs were found to have a rather poor survival rate. Mouse ESC derived MNs on the other hand survive well in transplantation paradigms involving embryonic and neonatal CNS, with their axons innervating muscles (Yohn et al., 2008). Likewise mouse ESC derived MNs can also survive when transplanted into adult mice, and their axons grow to denervated muscles (Deshpande et al., 2006). Yet, hESC-derived MNs transplanted into adult mice fail due to low survival (Lee et al., 2007). A recent study employing iPSC-derived neural progenitors for transplantation into rats, however, was able to report successful grafting and a high survival rate of transplanted cells and specification of grafted cells to MNs in the ventral horn of recipient animals (Popescu et al., 2013).

Despite mostly disappointing outcomes in approaches to graft human MNs in the CNS, the fact that mouse ESC-derived neurons, human iPSC-derived astrocytes, and recently even human neural progenitors can be grafted successfully bears hope for the transplantation of neurons. Strategies to achieve this will have to improve and promote neuron survival of either terminally differentiated neurons or committed neuronal precursors. In addition the grafted cells need to migrate long distances, extend neurites and axons to innervate their appropriate target muscles and make functional connections.

## ‘OMICS—Utilizing Big Data To Generate Disease Signatures

With a demographically growing number of documented cases of neurodegenerative diseases, the urgency to define the state and behavior of various CNS cells under homeostatic and diseased conditions is apparent. Nonetheless, our understanding of the CNS under diseased conditions such as neurodegeneration observed in ALS, Huntington’s disease and PD, despite dramatic progress over the past 3 decades, still is rather limited. Particularly with respect to ALS, while research using animal models has brought significant insight into some of the major events accompanying ALS, yet, not a single significant disease modifying therapy exists. With patient-derived iPSCs technology enabling the generation of CNS specific cell types, new avenues of studying disease progression and signatures in human cells can be explored.

The Library of Integrated Network-Based Cellular Signatures (LINCS) is a collaborative initiative with the aim to catalog cellular processes under conditions such as cell stress and disease. Funded by the National Institute of Health (NIH) the LINCS centers set out to collect large data sets of cellular events in human diseases compared to healthy control samples. Utilizing computational tools to integrate such diverse information into a comprehensive view of molecular disease events and the development of new biomarkers and therapeutics (Vidovic et al., 2014; Liu et al., 2015). That this approach is likely to reveal a wealth of new findings is supported by a line of previous LINCS studies in areas of cell lineage identity, genome modification and cancer research (Duan et al., 2014; Gujral et al., 2014; Ma’ayan and Duan, 2014; Olson et al., 2014; Fallahi-Sichani et al., 2015).

Specifically for neurodegenerative diseases the NeuroLINCS consortium has been established and plans to combine expertise in the fields of iPSC technology, disease modeling, whole genome sequencing, epigenomics, transcriptomics, proteomics, and metabolomics to integrate cell-based assays and high-throughput single-cell analysis with statistics, bioinformatics and computational biology. With regard to ALS this NeuroLINCS consortium would develop signatures of diseased motor neurons and glial cells under various ALS genotypes and baseline conditions. Eventually, we will determine how these patient genotypes in the diseased neurons interact with environmental perturbagens (glutamate and tunicamycin) to elicit responses related to mitochondrial and ER stress. Our understanding of the molecular circumstances of disease progression could be dramatically improved by the generation of quantitative molecular phenotypes and cell signatures of human neurons and glia, providing rational intervention points.

## Concluding Remarks

More than a century ago, Jean-Martin Charcot first described a disease entity of broad neurodegeneration known as ALS. Since then multiple molecular events associated with ALS disease progression have been identified, each of these working in concert leading to disease onset: (1) Developmental defects caused by genetic mutations making certain MN subtypes susceptible to aberrant maturation during CNS development could lead to early degeneration in adult life; (2) An inert susceptibility of mitochondria, provoked by an inability to balance ROS, making MNs more prone to cell stress and degeneration; (3) Misfolded proteins and protein-RNA aggregates triggering the ER UPR stress responses leading to initiation of MN apoptosis; and (4) The dysregulation of Ca^2+^ levels observed during disease progression is a common link to both mitochondrial and ER stress, as both organelles finely tune Ca^2+^ homeostasis. Particularly, the latter one provides putative insights into MN susceptibility through mitochondrial stress leading to increased local Ca^2+^ levels and ultimately functional alteration of ALS-critical proteins; given that Ca^2+^ itself is typically found to affect ALS-associated proteins and processes.

The post-mitotic MNs, however, are not the only cell types affected by the pathological processes and mutant genes involved in ALS. Glial cells, in fact, are functionally impaired, as shown in animal and stem cell models alike, and these ultimately impact MNs as well. Animal models and multidimensional “organs-on-a-chip” will be crucial when future studies are required to dissect CNS cell-cell interactions. However, animal models alone will not enable us to decipher the full complexity of an ALS patient’s phenotype. Years of research with animal models have led to mostly ineffective treatments for ALS. In this regard human models utilizing patient iPS cell technology continue to provide a wealth of additional insight into the relevant disease phenotype in pathophysiologically affected human cells. The logical next step will be to use both animal and cell models synergistically and coordinate collaborative efforts of large consortium studies such as the NeuroLINCS initiative. By utilizing ‘OMICS, longitudinal imaging, big data and machine-learning approaches on large cohort of ALS patient iPSC-derivatives harboring both familial and sporadic forms of the disease we may be able to discover central ALS-specific mechanistic signatures beyond the phenotype of an individual ALS mutation, and rather also across sporadic disease that affects almost 90% of the patients. Such an approach promises discovery of key biomarkers and pathways that shift subtype-specific motor neurons and glial cells out of balance. Whether iPS cell technology can fulfil such commitments remains to be determined.

Short-term approaches must develop early ALS biomarkers enabling faster patient evaluation and diagnosis. Mid-term goals are to improve MN survival and delay disease progression significantly, either by targeting MNs directly or indirectly via glial cells, or employing small molecules and neurotrophic factors to interfere with ROS production, Ca^2+^ deregulation and apoptotic signaling. Long-term strategies will require a detailed understanding of early signs of ALS initiation and the ability to repair and regenerate at least MN sets crucial for survival and extending life span. Recent reports (Popescu et al., 2013), provide a glimpse of hope to utilize patient-derived iPSCs for large scale screening of new diagnostic makers, and the perspective of regenerative cellular therapies to improve patients quality of life.

## Funding

This work was supported by Cedars-Sinai institutional startup funds (DS), National Center for Advancing Translational Sciences (NCATS), Grant UL1TR000124 (DS). DS is also supported by funds from National Institute of Neurological Disorders and Stroke (NINDS) grant U54NS091046. The funders had no role in study design, data collection and analysis, decision to publish, or preparation of the manuscript.

## Conflict of Interest Statement

The authors declare that the research was conducted in the absence of any commercial or financial relationships that could be construed as a potential conflict of interest.
